# Thrombotic Risk of Targeted Therapies in Breast Cancer: A Real-World Cohort Study

**DOI:** 10.21203/rs.3.rs-10813538/v1

**Published:** 2026-10-11

**Authors:** Wesley Boland, Gregory Guzik, Eduarda Grinsztejn, Jaime A Perez, Keri Plevniak, Bethany Samuelson Bannow, Lalitha Nayak, James Martin

**Affiliations:** University Hospitals Cleveland Medical Center; Indiana University School of Medicine; Weill Cornell Medicine; University Hospitals Clinical Research Center; University Hospitals Clinical Research Center; Cleveland Clinic; Indiana University School of Medicine; New York University

**Keywords:** Breast Cancer, Thrombosis, Targeted Therapy, Palbociclib, Real-world evidence

## Abstract

**Purpose:**

Targeted therapies for breast cancer, including CDK4/6, PARP, mTOR, and PI3K inhibitors, have raised concerns regarding thrombosis risk, but real-world data comparing these agents remain limited. We investigated thrombosis incidence and risk factors in a large national cohort of breast cancer patients.

**Methods:**

In this retrospective cohort study using the TriNetX national database, we evaluated the thrombotic risk and associated factors among patients with breast cancer treated with these therapies. The primary outcome was any arterial or venous thrombotic event.

**Results:**

Among 4,165 patients on targeted therapy, treatment with CDK4/6i was associated with increased risk of any thrombosis (HR 3.36, 95% CI 2.89–3.92) and venous thrombosis (HR 4.45, 95% CI 3.68–5.39). PARPi (HR 3.78, 95% CI 1.82–7.85; venous HR 5.79, 95% CI 2.52–13.3) and everolimus (HR 6.59, 95% CI 3.82–11.4; venous HR 9.67, 95% CI 5.29–17.7) showed similar associations. Within CDK4/6i, palbociclib carried the highest hazard for any thrombosis (HR 3.64, 95% CI 3.02–4.39) and venous thrombosis (HR 5.11, 95% CI 4.05–6.45). Nicotine dependence (HR 2.01, 95% CI 1.91–2.12), brain metastases (HR 4.20, 95% CI 2.98–5.93), and higher BMI (HR 1.03, 95% CI 1.03–1.03) were independent risk factors.

**Conclusion:**

This real-world analysis demonstrates that CDK4/6i and everolimus were associated with increased risk of both arterial and venous thrombosis, while PARPi was specifically associated with venous thrombosis. Similar risk was seen across CDK4/6 inhibitor agents. These results support risk stratification tools incorporating both agent and patient-level factors to better inform clinical management.

## Introduction

Breast cancer is the most common cancer diagnosed in women and the second leading cause of cancer-related mortality^1^. The landscape of breast cancer treatment has evolved significantly with the integration of targeted molecular therapies directed at specific oncogenic pathways, including pharmacologic inhibitors of cyclin-dependent kinases 4/6 (CDK4/6i), poly (ADP-ribose) polymerase (PARPi), mammalian target of rapamycin (mTOR), and phosphoinositide 3-kinase (PI3K). These agents are primarily used in advanced or metastatic setting. CDK4/6i (palbociclib, ribociclib, and abemaciclib) are commonly combined with endocrine therapy. mTORi, (everolimus), is used with exemestane following progression on non-steroidal aromatase inhibitor ^2^. PI3Ki (alpelisib) is used with fulvestrant in PIK3CA mutated cases ^3^. Lastly, PARPi, (olaparib and talazoparib), are indicated for patients with germline BRCA mutations ^4^. Following demonstrated benefit in the metastatic setting, abemaciclib, ribociclib, and olaparib have also gained Food and Drug Administration (FDA) approval for use in the adjuvant treatment of high-risk early breast cancer ^5^. Together, these agents represent major strides in precision oncology, tailoring treatment based on hormone receptor status, genetic mutations, and mechanisms of resistance and have improved progression-free survival and overall survival outcomes for many patients.

Thrombosis is a well-documented complication of cancer and a leading cause of mortality second only to disease progression ^6–8^. Patients with breast cancer receiving targeted therapies have been shown to be at heightened risk of thrombosis. CDK4/6i, specifically abemaciclib, have demonstrated elevated rates of thrombotic events in both clinical trials and real-world studies compared to control arms ^9–13^. Similarly, PARPi have been linked to an increased risk of thromboembolic events in patients with breast, ovarian, and prostate cancer ^14,15^. While data specifically on mTORi in breast cancer are scarce, prior studies have associated everolimus with thrombosis in other clinical contexts ^16,17^. For PI3Ki, alpelisib clinical trials have not reported thrombotic events, though real-world data remain limited ^3^. Beyond specific therapies, factors such as prior thrombotic events, metastatic disease, age, and obesity are recognized contributors to thrombosis risk in cancer patients ^18–20^. Yet, the interplay of these established risk factors with targeted therapies in breast cancer requires further investigation. This study aimed to leverage a large, national, real-world cohort to comprehensively assess the thrombotic risk in patients with breast cancer being treated with these targeted therapies and to elucidate the contributions of both drug- and patient-specific risk factors.

## Methods

### Data source and selection

We performed a retrospective cohort study using a de-identified national database with 85 contributing health care organizations (HCO), including over 129 million patients (TriNetX Research Network, Cambridge, MA; date of data access: April 25, 2024; IRB exempt)^21^. We identified all patients with a diagnosis of malignant neoplasm of the breast (International Classification of Diseases, Tenth Revision [ICD-10]: C50) on or after March 1st, 2012. Patients were excluded if they had a prior history of venous or arterial thrombosis or anticoagulation use prior to breast cancer diagnosis (Table S1). Two cohorts were compared, those with breast cancer and prescribed one of the drug classes of interest, CDK 4/6i (ribociclib, abemaciclib, or palbociclib), PARPi (olaparib or talazoparib), mTORi (everolimus), or PI3Ki (alpelisib) and those diagnosed with breast cancer and did not receive treatment with one of the drugs of interest. Medication start was defined as the first recorded date of each therapy plus a 7-day washout period, to conservatively attribute outcome to medication use. Medication discontinuation was defined as 60 days after the last recorded date of targeted therapy.

The primary outcome was thrombosis occurring at least 1 day after breast cancer diagnosis. Thrombotic events were defined as either arterial (myocardial infarction, arterial embolism and thrombosis, and cerebral infarction) or venous (venous embolism and pulmonary embolism) (Table S1). To improve specificity, a venous thromboembolism (VTE) diagnosis was required to be accompanied by an anticoagulant prescription (Table S1) issued between the date of diagnosis and 365 days thereafter. Thrombotic outcomes were assessed in aggregate and separately by type. Patients were followed from breast cancer diagnosis to the first thrombotic event or were censored at last encounter or death. Additional covariates investigated included age, BMI, nicotine dependence, and brain metastases. Metastatic disease was defined by the presence of ICD-10 codes for secondary malignant neoplasms (C78 and C79).

### Statistical analysis

Baseline differences were calculated using independent t-test for continuous variables and chi-square or Fisher’s exact test for categorical variables. A time-dependent Cox proportional hazards model was used to assess the relationship between thrombosis and targeted therapy, with targeted therapy treated as a time-varying covariate. Covariates included nicotine dependence, brain metastases, concomitant endocrine therapy (tamoxifen, fulvestrant, exemestane, letrozole, or anastrozole), age at breast cancer diagnosis, and BMI. The reference group for all models was patients with breast cancer not receiving any targeted therapy. Estimates of hazard ratios (HR) and 95% confidence intervals (CI) were used to describe the hazard of thrombosis. P-values of α < 0.05 were considered statistically significant. Sub-analyses were performed in three populations: patients with metastatic disease receiving targeted therapy, patients receiving CDK4/6i, and patients with metastatic disease receiving CDK4/6i.

### Ethics

Human Ethics and Consent to Participate declarations: not applicable. This study used a publicly available dataset and was deemed an IRB-exempt study. University Hospitals IRB provided approval on 26 April 2023 (Study# 20221392). This project was supported by the Clinical and Translational Science Collaborative of Northern Ohio which is funded by the National Institutes of Health, National Center for Advancing Translational Sciences, Clinical and Translational Science Award grant, UM1TR004528. The content is solely the responsibility of the authors and does not necessarily represent the official views of the NIH.

## Results

Our study included 155,281 patients with a diagnosis of breast cancer, of whom 4,165 were taking one of the medications of interest during the study period. In the targeted therapy group, the mean age was 58.1 ± 13.0 years old and was predominantly white (72.4%) (Table 1). CDK4/6i were overwhelmingly the most prescribed, with 82.1% of patients receiving one of these medications. Specifically, palbociclib was used by 47.9% of patients, followed by abemaciclib (24.9%) and ribociclib (9.3%). Other therapies included everolimus (9.5%), alpelisib (1.1%), olaparib (7.2%), and talazoparib (0.2%). The most commonly prescribed concomitant endocrine therapy amongst those on targeted therapy was anastrozole (34.0%), followed by fulvestrant (29.8%), letrozole (28.8%), exemestane (18.8%), and tamoxifen (17.7%). Among patients on targeted therapy, 4.2% had a coded diagnosis of metastatic disease, including 0.4% with brain metastases (Table 1).

A total of 10,380 thrombotic events were observed: 10,168 in patients not on targeted therapy and 212 in patients on targeted therapy. In the primary time-dependent Cox model, any targeted therapy was associated with an increased hazard of thrombosis compared with no targeted therapy (HR 3.64; 95% CI 3.15–4.20; p < 0.001) after adjustment (Table S2). When analyzed by class, CDK4/6i (HR 3.36, 95% CI 2.89–3.92), PARPi (HR 3.78, 95% CI 1.82–7.85), and everolimus (HR 6.59, 95% CI 3.82–11.4) were each associated with a significantly increased risk of any thrombosis (all p < 0.001) after adjustment for concomitant endocrine therapy (Table 2, Fig. 1). Estimates were similar in the model without adjustment for concomitant endocrine therapy (Table S3). Alpelisib was not included in the regression models due to an insufficient number of events.

Risk varied by thrombosis type. For venous thrombosis, CDK4/6i (HR 4.45, 95% CI 3.68–5.39), PARPi (HR 5.79, 95% CI 2.52–13.3), and everolimus (HR 9.67, 95% CI 5.29–17.7) were each associated with a significantly increased risk (all p < 0.001) (Table 2). For arterial thrombosis, CDK4/6i (HR 2.50, 95% CI 1.97–3.18, p < 0.001) and everolimus (HR 3.20, 95% CI 1.01–10.1, p = 0.047) were significantly associated, whereas PARPi was not (HR 1.92, 95% CI 0.47–7.84, p = 0.365) (Table 2).

Concomitant endocrine therapy was associated with an increased hazard of any thrombosis (HR 1.12, 95% CI 1.08–1.16, p < 0.001) and venous thrombosis (HR 1.27, 95% CI 1.20–1.35, p < 0.001), but not arterial thrombosis (HR 1.02, 95% CI 0.96–1.07, p = 0.56). Each established risk factor was independently associated with thrombosis: nicotine dependence (HR 2.01; 95% CI 1.91–2.12), brain metastasis (HR 4.20; 95% CI 2.98–5.93), older age at diagnosis (HR 1.08 per year; 95% CI 1.08–1.09), and higher BMI (HR 1.03 per unit; 95% CI 1.03–1.03); all p < 0.001 (Table 2).

Within CDK4/6i, palbociclib was significantly associated with an increased risk of any thrombosis (HR: 3.64, 95% CI: 3.02–4.39, p < 0.001), venous thrombosis (HR: 5.11, 95% CI: 4.05–6.45, p < 0.001), and arterial thrombosis (HR: 2.64, 95% CI: 1.98–3.53, p < 0.001) (Table 3).

Abemaciclib was similarly associated with increased risk of any thrombosis (HR: 3.06, 95% CI: 2.22–4.22, p < 0.001), venous thrombosis (HR: 3.68, 95% CI: 2.46–5.51, p < 0.001), and arterial thrombosis (HR: 2.44, 95% CI: 1.48–4.03, p < 0.001). Ribociclib was significantly associated with any thrombosis (HR: 2.70, 95% CI: 1.66–4.37, p < 0.001) and venous thrombosis (HR: 3.44, 95% CI: 1.92–6.16, p < 0.001), but did not reach significance for arterial thrombosis (HR: 1.85, 95% CI: 0.83–4.10, p = 0.130), however n = 6 (Table 3). Baseline characteristics for the CDK4/6i cohorts were similar to the overall cohort (Table S4).

In a subgroup analysis of patients with a diagnosis of metastatic cancer, CDK4/6i use was not significantly associated with increased thrombosis risk compared to no targeted therapy across any outcome. In the overall targeted therapy vs. no targeted therapy comparison, CDK4/6i was not significantly associated with any thrombosis (HR: 0.61, 95% CI: 0.27–1.37, p = 0.227), venous thrombosis (HR: 0.70, 95% CI: 0.25–1.98, p = 0.508), or arterial thrombosis (HR: 0.46, 95% CI: 0.12–1.77, p = 0.262) (Tables S5). Ribociclib had insufficient events for modeling, limiting analysis to palbociclib and abemaciclib, neither of which reached significance for any thrombotic outcome (Table S6). These findings should be interpreted with caution given small event counts; comparative analysis of PARPi, mTOR, and PI3K inhibitors was not possible due to insufficient outcome events.

## Discussion

This study used a large, real-world database to investigate the risk of thrombosis associated with targeted therapies in breast cancer. Our analysis is consistent with prior findings that certain classes of targeted agents, including CDK4/6i, PARPi, and everolimus, are each associated with an increased incidence of venous thrombosis. However, our findings challenge previous reports regarding the relative thrombotic risk of individual CDK4/6i agents, suggesting a more complex relationship than previously understood.

Trials leading to the FDA approval of CDK4/6i showed an increased risk of VTE compared to the control arms with rates ranging from 0.6 to 6.1%. Subsequent meta-analyses and real-world studies consistently reported elevated VTE rates as high as 10% ^9,10,22^. One meta-analysis found that the VTE risk was more than doubled in patients with breast cancer taking CDK4/6i (OR 2.70 [95% CI: 2.00, 3.63], p < 0.001) ^11^. Within this class, abemaciclib was reported to have the highest incidence (OR 4.4) compared to palbociclib (OR 2.35), a finding potentially explained by in vitro evidence linking abemaciclib to platelet activation ^11^. The trials leading to abemaciclib approval, MONARCH 2 and 3, reported substantially higher rates of VTE in the abemaciclib arms (4.8% and 6.1%, respectively) compared to the placebo arms (< 1%) ^5,23,24^. One observational study found that arterial events accounted for roughly one-third of the total thrombotic events in patients treated with CDK4/6i, emphasizing that while venous events predominate, the arterial component is not negligible ^13^. Furthermore, patients who develop thrombosis on these therapies experience a higher risk of mortality than those who did not ^12,13^.

We found that patients taking a CDK4/6i had a higher risk of both venous and any thrombosis, aligning with existing literature showing a clinically meaningful prothrombotic class effect of these therapies ^25–27^. Notably, the magnitude of risk observed in our cohort exceeded that reported in prior studies, with hazard ratios for venous thrombosis ranging from 3.44 to 5.11 across individual agents. Contrary to findings from other cohorts, the risk of thrombosis observed with palbociclib and abemaciclib was similar. While the pathophysiological mechanisms linking these agents to thrombotic events remain incompletely understood, their clinical association with thrombotic risk demands attention. Abemaciclib specifically has been linked to platelet activation and increased thrombotic tendency, supported by in vitro evidence showing it uniquely stimulates collagen-induced platelet activation (via P-selectin expression) compared to palbociclib or ribociclib, offering a mechanistic basis for its thrombotic risk ^11^. Ribociclib demonstrated a predominantly venous thrombotic signal (HR 3.44) without a significant arterial association, suggesting possible mechanistic heterogeneity within the CDK4/6i class. Of note, palbociclib and abemaciclib were significantly associated with arterial thrombosis, an observation that extends beyond the predominantly venous signal emphasized in prior literature and underscores that arterial events represent a clinically meaningful component of CDK4/6i-associated thrombotic risk.

We found that mTOR inhibition with everolimus was associated with a significant increase in the risk of venous thrombosis (HR 9.67) and any thrombosis (HR 6.59). To our knowledge, no published literature specifically addresses the thrombosis risk associated with mTORi in breast cancer or other malignancies. While everolimus has been associated with a procoagulant state in renal transplant recipients, potentially through endothelial dysfunction, it remains unclear whether these findings are applicable to patients with breast cancer, given the distinct patient populations and therapeutic indications ^16,17^. Our finding of an increased thrombotic risk with everolimus in breast cancer highlights a critical gap in the literature and warrants further investigation.

PARPi were associated with a significantly increased risk of venous thrombosis (HR 5.79) and any thrombosis (HR 3.78), substantially exceeding prior estimates in patients with solid tumors (OR 1.49, P = 0.004) ^14^ and prostate cancer specifically (OR 1.98, 95% CI: 1.06–3.70, P = 0.03) ^15^, suggesting the thrombotic burden of these agents in a real-world breast cancer population may be considerably greater than previously appreciated. Given the limited data on PARPi-associated thrombosis specifically in breast cancer, this real-world evidence is particularly valuable. PARPi were not significantly associated with arterial thrombosis, though this may reflect limited event counts among exposed patients. Regarding PI3Ki, Alpelisib could not be evaluated in regression models due to insufficient outcome events, consistent with clinical trial data reporting no thrombotic events in the experimental arm^3^. Among patients with metastatic breast cancer, CDK4/6i use was not significantly associated with any thrombotic outcome. These analyses were substantially limited by small sample size and event counts, precluding evaluation of PARPi, everolimus, and alpelisib in this context.

Beyond therapy-specific risks, intrinsic risk factors, including smoking and higher BMI, contributed to thrombosis risk in our cohort. The role of obesity as an independent risk factor for thrombosis remains inconsistent in the literature; while some studies link a BMI > 25 or > 30 to increased VTE risk, this was not observed specifically in patients on CDK4/6i ^13,20,28–30^. Smoking has been broadly associated with an increased VTE risk in patients with active cancer, though direct associations in breast cancer, particularly among patients receiving chemotherapy or CDK4/6i, have been inconsistent. ^13,18,31^. A prospective study of patients with breast or gastrointestinal cancer receiving adjuvant chemotherapy suggested a two-fold increased risk of VTE among smokers (former and current), though this finding did not reach statistical significance ^19^. Brain metastases were associated with increased thrombosis risk, concordant with literature indicating their role as an independent risk factor for VTE ^20^. Lastly, concomitant endocrine therapy was associated with an increased hazard of any thrombosis and venous thrombosis, but not arterial thrombosis, consistent with the known prothrombotic effects of these hormonal agents. These findings underscore the multifactorial nature of thrombosis in cancer patients, emphasizing that effective risk stratification must integrate both therapy-specific and patient-specific factors. While primary thromboprophylaxis is not justified in all patients, identifying high-risk individuals is increasingly important ^32^. The Khorana score, a widely used risk stratification tool for cancer-associated thrombosis, does not currently account for exposure to targeted therapies ^33^. Given the high thrombotic burden observed in our study, integrating additional risk factors into clinical decision-making may help guide anticoagulation considerations.

Our real-world dataset highlights the need for prospective studies evaluating thrombosis rates, anticoagulation strategies, and long-term vascular outcomes in this population. Real-world populations are inherently more heterogeneous, often being older and having a higher prevalence of pre-existing risk factors. As targeted therapies become more widely used in breast cancer treatment, a standardized approach to evaluating thrombosis risk prior to initiation of therapy is warranted. This may include incorporating routine screening for thrombotic history, BMI, and smoking status into treatment decision-making. In clinical practice, oncologists should maintain a heightened awareness of thrombotic complications associated with targeted therapies, ensuring close monitoring and a multidisciplinary approach to risk management.

Our study has several limitations. As a retrospective analysis of electronic health record data, the potential for missing or misclassified data persists, particularly the underrepresentation of secondary malignant neoplasm coding. However, TriNetX has been widely utilized in real-world research, demonstrating its acceptability for observational studies ^34^. Thrombotic events were identified using ICD-10 codes and an anticoagulant prescription, which limits our ability to distinguish symptomatic versus asymptomatic cases or determine whether these events required hospitalization or intervention. Confounding by indication may persist despite the use of time-dependent covariate modeling, particularly in the metastatic subgroup, and residual confounding by performance status and surveillance intensity may have contributed to the observed associations. Relatedly, patients with perceived thrombotic susceptibility may have been preferentially prescribed palbociclib as the presumed lower-risk agent, concentrating high-risk individuals within that exposure group and potentially explaining its divergence from prior studies. Furthermore, nicotine dependence was used as a proxy for smoking history and may not fully capture the impact of smoking on thrombotic risk. Despite these limitations, the large sample size and multi-institutional nature of our cohort enhance the generalizability of our findings. Nevertheless, prospective studies are needed to validate these results, refine risk stratification models, and evaluate the potential role of thromboprophylaxis in this high-risk population.

## Conclusion

In this large, real-world cohort of breast cancer patients, treatment with CDK4/6i, PARPi, and mTORi was each independently associated with a significantly increased risk of any thrombosis, with the strongest signal observed for venous thrombosis. Within the CDK4/6i class, thrombosis risk was of similar magnitude between agents, arguing against a clear intra-class difference in risk. Alongside these therapy-specific associations, traditional risk factors, including nicotine dependence, brain metastases, older age, and higher BMI, were independently associated with thrombosis. Consequently, these findings highlight the necessity for improved risk stratification models that incorporate both the choice of specific targeted agent and baseline patient characteristics for a more accurate assessment of thrombotic risk.

## Supplementary Material

This is a list of supplementary files associated with this preprint. Click to download.

- ThrombosisSupplementV220260701.docx

## Figures and Tables

**Figure 1:**
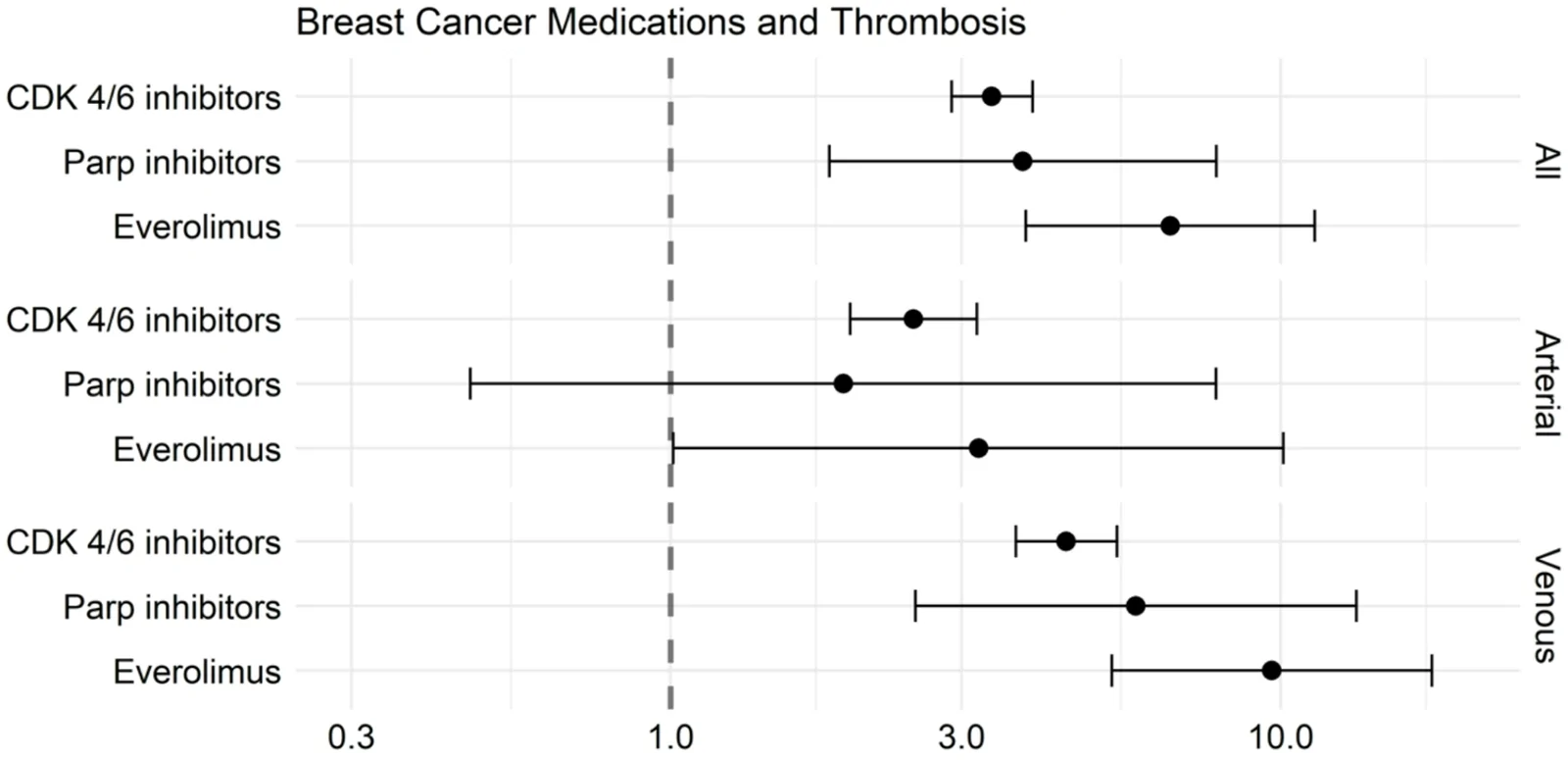
Forest plot of Hazard ratios for targeted therapy mediations. Figure shows hazard ratios and 95% CI for targeted therapies CDK 4/6i, PARPi, and Everolimus for the outcomes of any thrombosis, arterial thrombosis and venous thrombosis.

**Table 1.** Baseline characteristics between those that have breast cancer and are not taking a targeted therapy and those that are taking a targeted therapy. Significant p-values in bold.

Characteristic	Overall^1^ N = 155,281	No Treatment with medication^1^ N = 151,116	Treatment with medication^1^ N = 4,165	p-value^2^
**Age at diagnosis**	60.5 ± 12.6	60.6 ± 12.6	58.1 ± 13.0	**<0.001**
**Race**				**<0.001**
American Indian or Alaska Native	371 (0.2%)	365 (0.2%)	6 (0.1%)	
Asian	5,588 (3.6%)	5,449 (3.6%)	139 (3.3%)	
Black or African American	17,065 (11.0%)	16,545 (10.9%)	520 (12.5%)	
Native Hawaiian or Other Pacific Islander	213 (0.1%)	200 (0.1%)	13 (0.3%)	
Other Race	5,654 (3.6%)	5,458 (3.6%)	196 (4.7%)	
Unknown	16,009 (10.3%)	15,735 (10.4%)	274 (6.6%)	
White	110,381 (71.1%)	107,364 (71.0%)	3,017 (72.4%)	
**Ethnicity**				**<0.001**
Hispanic or Latino	10,503 (6.8%)	10,167 (6.7%)	336 (8.1%)	
Not Hispanic or Latino	128,499 (82.8%)	124,970 (82.7%)	3,529 (84.7%)	
Unknown	16,279 (10.5%)	15,979 (10.6%)	300 (7.2%)	
**Nicotine Dependence**	14,845 (9.6%)	14,295 (9.5%)	550 (13.2%)	**<0.001**
**Breast Cancer Medications**				**--**
CDK4/6i	3,418 (2.2%)	0 (0.0%)	3,418 (82.1%)	
PARPi	306 (0.2%)	0 (0.0%)	306 (7.3%)	
everolimus	396 (0.3%)	0 (0.0%)	396 (9.5%)	
alpelisib	45 (0.0%)	0 (0.0%)	45 (1.1%)	
**cdk4_6**				
ribociclib	386 (0.2%)	0 (0.0%)	386 (9.3%)	**<0.001**
palbociclib	1,995 (1.3%)	0 (0.0%)	1,995 (47.9%)	**<0.001**
abemaciclib	1,037 (0.7%)	0 (0.0%)	1,037 (24.9%)	**<0.001**
**parp**				
olaparib	299 (0.2%)	0 (0.0%)	299 (7.2%)	**<0.001**
talazoparib	7 (0.0%)	0 (0.0%)	7 (0.2%)	**<0.001**
**Comorbidities**				
**Metastatic Cancer**	1,686 (1.1%)	1,511 (1.0%)	175 (4.2%)	**<0.001**
**Malignant neoplasm of brain**	290 (0.2%)	275 (0.2%)	15 (0.4%)	**0.009**
**Lab results**				
**BMI**	28.3 ± 6.7	28.2 ± 6.7	28.8 ± 6.6	**<0.001**
**Hemoglobin**	13.2 ± 1.6	13.2 ± 1.6	13.1 ± 1.9	**0.002**
Unknown	84,690	82,463	2,227	
**Concomitant medications**				
anastrozole	30,546 (19.7%)	29,129 (19.3%)	1,417 (34.0%)	
exemestane	8,858 (5.7%)	8,076 (5.3%)	782 (18.8%)	
fulvestrant	2,165 (1.4%)	922 (0.6%)	1,243 (29.8%)	
letrozole	16,508 (10.6%)	15,310 (10.1%)	1,198 (28.8%)	
tamoxifen	21,460 (13.8%)	20,722 (13.7%)	738 (17.7%)	
More than 1 concomitant medication				
No concomitant medication	95,538 (61.5%)	94,721 (62.7%)	817 (19.6%)	
**elacestrant**	24 (0.0%)	2 (0.0%)	22 (0.5%)	**<0.001**

1 Mean ± SD; n (%)

2 Welch Two Sample t-test; Pearson’s Chi-squared test; Fisher’s exact test

**Table 2.** Table presents results from time-dependent Cox proportional hazard models for the outcome of thrombosis comparing those that had breast cancer and are not taking medication and those that are taking medication, including concomitant medication as a covariate. Table presents hazard ratios and 95% confidence intervals with significant p-values in bold.

	Any Thrombosis		
Characteristic	HR^1^	95% CI^1^	p-value
**Medication**			
CDK4/6i	3.36	2.89, 3.92	**<0.001**
PARPi	3.78	1.82, 7.85	**<0.001**
Everolimus	6.59	3.82, 11.4	**<0.001**
**Nicotine dependence**	2.01	1.91, 2.12	**<0.001**
**Malignant neoplasm of brain**	4.20	2.98, 5.93	**<0.001**
**Age**	1.08	1.08, 1.09	**<0.001**
**BMI**	1.03	1.03, 1.03	**<0.001**
**Concomitant medication**	1.12	1.08, 1.16	**<0.001**

1 HR = Hazard Ratio, CI = Confidence Interval; Reference level for medication (No targeted therapy), Nicotine dependence (No), Malignant neoplasm of brain

**Table 3.** Thrombosis outcome for patients with breast cancer comparing those who are on CDK 4/6i and those who are not taking CDK4/6i medication. Table presents results from time-dependent Cox proportional hazard models, including concomitant medication as a covariate, showing hazard ratios and 95% confidence intervals with significant p-values in bold.

	Any Thrombosis		
Characteristic	HR^1^	95% CI^1^	p-value
**Medication**			
abemaciclib	3.06	2.22, 4.22	**<0.001**
palbociclib	3.64	3.02, 4.39	**<0.001**
ribociclib	2.70	1.66, 4.37	**<0.001**
**Nicotine dependence**	2.03	1.93, 2.13	**<0.001**
**Malignant neoplasm of brain**	4.29	3.02, 6.10	**<0.001**
**Age**	1.08	1.08, 1.09	**<0.001**
**bmi**	1.03	1.03, 1.03	**<0.001**
**Concomitant medication**	1.12	1.08, 1.16	**<0.001**

1 HR = Hazard Ratio, CI = Confidence Interval; Reference level for medication (No CDK 4/6i), Nicotine dependence (No), Malignant neoplasm of brain (No), C
